# Neurodetector: EEG-Based Cognitive Assessment Using Event-Related Potentials as a Virtual Switch †

**DOI:** 10.3390/brainsci15090931

**Published:** 2025-08-27

**Authors:** Ryohei P. Hasegawa, Shinya Watanabe

**Affiliations:** 1 Research Institute on Human and Societal Augmentation (RIHSA), National Institute of Advanced Industrial Science and Technology (AIST), 1-1-1 Umezono, AIST Tsukuba Headquarters, Tsukuba 305-8560, Ibaraki, Japan; 2 Faculty of Engineering, University of Fukui, 3-9-1 Bunkyo, Fukui 910-0017, Fukui, Japan; 3 Innovative Research Center for Preventive Medical Engineering, Nagoya University, Furocho NIC, Chikusa, Nagoya 464-0819, Aichi, Japan; 4 Parallel Brain Interaction Sensing Division, Tokyo University of Science, 2641 Yamazaki, Noda 278-8510, Chiba, Japan; 5 Department of Neurosurgery, Mito Kyodo General Hospital, Tsukuba University Hospital Mito Area Medical Education Center, Mito 310-0015, Ibaraki, Japan; 6 Institute of Medicine, University of Tsukuba, Tsukuba 305-8576, Ibaraki, Japan

**Keywords:** BCI, EEG, event-related potentials, cognitive assessment

## Abstract

**Background/Objectives:** Motor decline in older adults can hinder cognitive assessments. To address this, we developed a brain–computer interface (BCI) using electroencephalography (EEG) and event-related potentials (ERPs) as a motor-independent EEG Switch. ERPs reflect attention-related neural activity and may serve as biomarkers for cognitive function. This study evaluated the feasibility of using ERP-based task success rates as indicators of cognitive abilities. The main goal of this article is the development and baseline evaluation of the Neurodetector system (incorporating the EEG Switch) as a motor-independent tool for cognitive assessment in healthy adults. **Methods:** We created a system called Neurodetector, which measures cognitive function through the ability to perform tasks using a virtual one-button EEG Switch. EEG data were collected from 40 healthy adults, mainly under 60 years of age, during three cognitive tasks of increasing difficulty. **Results:** The participants controlled the EEG Switch above chance level across all tasks. Success rates correlated with task difficulty and showed individual differences, suggesting that cognitive ability influences performance. In addition, we compared the pattern-matching method for ERP decoding with the conventional peak-based approaches. The pattern-matching method yielded a consistently higher accuracy and was more sensitive to task complexity and individual variability. **Conclusions:** These results support the potential of the EEG Switch as a reliable, non-motor-dependent cognitive assessment tool. The system is especially useful for populations with limited motor control, such as the elderly or individuals with physical disabilities. While Mild Cognitive Impairment (MCI) is an important future target for application, the present study involved only healthy adult participants. Future research should examine the sources of individual differences and validate EEG switches in clinical contexts, including clinical trials involving MCI and dementia patients. Our findings lay the groundwork for a novel and accessible approach for cognitive evaluation using neurophysiological data.

## 1. Introduction

### 1.1. Background and Clinical Context

In recent years, in the field of preventive medicine, increasing attention has been paid to identifying and addressing frailty, a state of physical, mental, and social vulnerability that often precedes the need for nursing care, and to supporting individuals who have already entered this stage [1,2]. Mild Cognitive Impairment (MCI), a precursor to dementia that is becoming increasingly prevalent in developed countries, is regarded as a form of “cognitive frailty” [3]. While no established effective disease-modifying treatment currently exists to completely halt or reverse the progression of dementia, research has shown that early detection of MCI and appropriate interventions can help prevent progression to dementia [4]. MCI represents a transitional stage between normal aging and dementia, and is characterized by a slight but noticeable decline in cognitive function. Although 14–40% of individuals with MCI eventually return to normal cognitive function [5,6,7,8,9], many others exhibit a persistent form of MCI that does not progress to dementia [8,10], while epidemiological studies have shown that approximately 10–15% of individuals with MCI progress to dementia each year, corresponding to a cumulative conversion rate of about 20–40% within three years [11,12].

Traditional neuropsychological assessments often rely on verbal or physical responses [13,14], which may pose challenges to elderly individuals with motor impairments. For such populations, cognitive assessment methods that are independent of motor functions are necessary. Objective diagnostic tools, such as volumetric magnetic resonance imaging (MRI), positron emission tomography (PET), and molecular imaging (amyloid and tau PET), have proven useful in diagnosing MCI [15,16]. However, owing to their high cost, invasiveness, and impracticality for repeated use, these methods may not be ideal for routine clinical screening. In contrast, EEG is a promising alternative, offering a cost-effective, non-invasive, and widely available method for assessing brain function [17,18,19]. It should be noted that although MCI is highlighted here as an important background and potential application field, the present study did not include participants with MCI; rather, it focused on healthy adults to establish baseline feasibility.

### 1.2. Related Works and Challenges in ERP-Based Cognitive Assessment

Event-related potentials (ERPs) have long been investigated as neurophysiological indicators of cognitive function and decline [20]. ERPs are small voltage fluctuations in electroencephalography that are synchronized with external sensory or cognitive events and often originate in the parietal lobe [21]. ERPs are considered the superimposition of multiple component waveforms, each reflecting sensory, cognitive, or motor processes or their interactions. Among these, the P300 component has received particular attention as a prominent and typically the largest component within ERP waveforms, serving as a reliable marker of cognitive function [22]. This component is characterized by a positive deflection that occurs approximately 300 ms after stimulus presentation, and is generally associated with attentional resource allocation and working memory updating.

The latency and amplitude of the P300 have been proposed as indicators of cognitive status, with latency and amplitude decreasing during normal aging [22,23,24,25], and more pronounced changes occurring in dementia or MCI [26,27,28]. However, such changes typically require averaging across large groups, which limits their diagnostic power in single-subject assessments [29,30,31].

A major limitation is the substantial inter-individual variability in ERP waveforms [20,32], which hinders clinical interpretation but is counterbalanced by the stability of these features within individuals, a property exploited in biometric identification [33,34,35,36].

To improve single-subject applicability, recent approaches have explored multivariate analyses and machine learning to exploit spatiotemporal patterns beyond traditional peak-based metrics [37,38].

### 1.3. BCI-Based Approaches for Cognitive Evaluation

In response to the need for communication and cognitive assessment methods that do not rely on verbal or motor functions, brain–machine interface (BMI) technology has emerged as a promising alternative for individuals with severe communication and motor impairments. Brain–machine interfaces are systems that enable direct communication between the brain and external devices and are often used to restore or augment motor function. While early BMI research focused primarily on invasive approaches using intracortical electrodes for motor control applications [39], non-invasive alternatives, such as EEG-based BCIs, have gained traction for their potential in enabling communication and cognitive assessment without the need for surgical intervention [40,41]. In this context, BCIs are typically considered a non-invasive subclass of BMIs. Among these, the P300 component has been extensively utilized, most notably in the “P300 speller,” a virtual typewriter that enables communication through brain responses elicited by focusing on visual stimuli [42]. The clinical applicability of such P300-based systems has been actively explored, especially for individuals with severe motor impairments such as amyotrophic lateral sclerosis (ALS) [43,44].

Although P300-based systems have shown promise in controlled laboratory settings, their translation into real-world environments, particularly in caregiving contexts, remains limited. This delay is largely attributed to practical issues such as signal variability, the need for frequent calibration, and user fatigue, all of which hinder stable and reliable operation during daily use [45,46,47,48]. Furthermore, performance can vary depending on modality (e.g., visual, auditory, or tactile) [49], cognitive status [50], and environmental factors. Even when used at home, the sustained performance of BCI systems in patients with ALS has shown variability, underscoring the importance of adaptive interfaces [51]. Importantly, the success of home-based BCI applications often depends on the caregivers responsible for tasks such as system setup, maintenance, and monitoring. However, their perspectives and operational burden are often underrepresented in BCI research. Studies have increasingly emphasized that caregiver engagement is crucial for the sustained use of BCI technologies and should be a key consideration in system design [47,52,53].

Our group has previously developed the Neurocommunicator^®^, a practical P300-based BCI for basic communication needs in patients with severe motor impairments [54]. In this system, users select one of eight pictograms (representing medical or care-related needs) presented in a 3 × 3 matrix by attending to a flashing target. The device integrated compact wireless EEG headgear, an adaptive stimulus presentation algorithm, and a hierarchical selection interface, enabling up to 512 selectable items. During clinical use, we observed that some users, particularly elderly or long-term bedridden patients, exhibited very weak ERP responses, suggesting possible cognitive decline that could not be assessed with standard neuropsychological tests. Similar performance variability has been reported in ALS populations depending on stimulus modality [49], attentional capacity [50], and long-term home use conditions [51]. These observations motivated the development of the Neurodetector, a system designed to assess cognitive ability using BCI principles, potentially benefiting both motor-impaired patients and broader at-risk populations [55].

### 1.4. Objectives and Contributions of This Research

To address the practical limitations discussed above, we aimed to develop a cognitive assessment system, called the Neurodetector, which can be used without overt motor responses, is operated by trained clinical or technical staff, and is robust across individual differences in ERP characteristics. To achieve this, we introduce the concept of the EEG Switch, a virtual selection mechanism that detects a user’s cognitive intent to select or respond by analyzing specific ERPs, particularly the P300 component. When a stimulus elicits a recognizable ERP pattern, the system interprets it as a selection—or “switching”—signal, enabling the user to make choices or perform tasks without physical movement.

This mechanism builds upon core technologies from our previous system, the Neurocommunicator, and introduces a novel brain–computer interface component referred to as the EEG Switch (formerly known as the Mind Switch) [54,55]. It supports BCI-based interactions by detecting whether a user has recognized or attended to a stimulus, effectively functioning as a “virtual response mechanism” for individuals who cannot communicate through conventional means.

Importantly, while the present study was conducted in healthy adults to establish baseline feasibility and evaluate attentional performance, the primary purpose of this paper is technological development and pilot validation of the Neurodetector system (with the EEG Switch) for motor-independent cognitive assessment. The overarching objective is to develop this technology for the early detection of mild cognitive impairment (MCI). Collecting robust normative data in healthy populations is critical to enable future comparative studies and clinical assessments targeting at-risk groups.

The primary objective of this study was to examine whether the EEG Switch can serve as an alternative, motor-independent measure of cognitive function, particularly top-down attention, which is essential for daily life activities such as following conversations in noisy environments or concentrating on a specific task while ignoring distractions [56]. In the context of BCI, the ability to intentionally modulate attention is also a key determinant of system performance, particularly in paradigms involving ERP components such as the P300 [57]. To this end, we conducted a pilot experiment to evaluate the performance characteristics of an EEG Switch in a series of cognitive tasks. Specifically, we tested the following hypotheses:

(1) The mean and distribution of success rates for cognitive tasks using the EEG Switch exceed the chance-level performance while avoiding ceiling effects.
(2) The proposed pattern-matching method, designed to accommodate individual differences in ERP responses, achieves higher success rates than the conventional peak-based analysis.
(3) The success rate of the EEG Switch is higher for control tasks that rely primarily on top-down attention and lower for tasks requiring broader cognitive processing.

Some of the data included in this paper were previously presented in abstract form (in Japanese) exclusively to attendees of the 64th Annual Meeting of the Japanese Society for Medical and Biological Engineering (JSMBE) [58].

The present paper extends these preliminary findings by providing a full methodological description, detailed statistical analyses, and novel multi-task comparisons that were not included in the conference abstract. The novelty of this study lies in demonstrating, for the first time, that a commercially available Neurodetector system, when combined with a customized pattern-matching approach, can quantify cognitive performance differences across multiple attentional tasks in healthy adults—thus establishing a baseline for future clinical application.

The remainder of this paper is organized as follows: Section 2 describes the experimental setup and analytical methods; Section 3 presents ERP and decoding results; Section 4 discusses implications, limitations, and future directions; and Section 5 concludes the study.

## 2. Materials and Methods

### 2.1. Participants

Forty volunteers (11 males and 29 females; mean age ± standard deviation: 43.0 ± 11.1 years) participated in the experiment. None of the participants had been diagnosed with dementia or MCI at the time of the study. Although the ultimate goal of this study was to develop a practical evaluation technique for elderly individuals at risk for dementia (typically defined as those aged 60 years or older globally [59] and 65 years or older in Japan [60]), the present study focused on collecting baseline experimental data from healthy adults aged 19–65 years, including only one participant aged 65 years and the remaining participants aged 59 years or younger.

The experimental protocol adhered to the Ethical Principles of the Declaration of Helsinki and the Ethical Guidelines for Medical Research Involving Human Subjects issued by the Ministry of Health, Labour, and Welfare of Japan. This study was approved by the Ergonomics Experimental Committee of our organization (AIST; Protocol ID: E2020-141A). Before participation, all participants received a full explanation of the study and provided their written informed consent.

### 2.2. Experimental Procedure

In this study, we developed a prototype of an EEG-based cognitive assessment system, the Neurodetector. This system integrates three core components: (1) a structured battery of cognitive tasks, including a top-down *Target-selection* task and two control conditions; (2) EEG signal acquisition using a custom headgear; and (3) single-trial ERP decoding through a virtual response mechanism called the *EEG Switch*. Section 2.3, Section 2.4 and Section 2.5 provide detailed descriptions of each of these components.

During the experiment, the participants were seated comfortably in front of a monitor and wore the EEG headgear. Instructions for each task were displayed visually, and participants were instructed to respond covertly (without any motor action), relying on their internal attention and intention. EEG signals were recorded continuously during task execution and responses were detected in real time via the *EEG Switch* mechanism. Each session consisted of multiple trials across different task types, lasting approximately 25–30 min in total.

### 2.3. Cognitive Tasks

**Figure 1:**
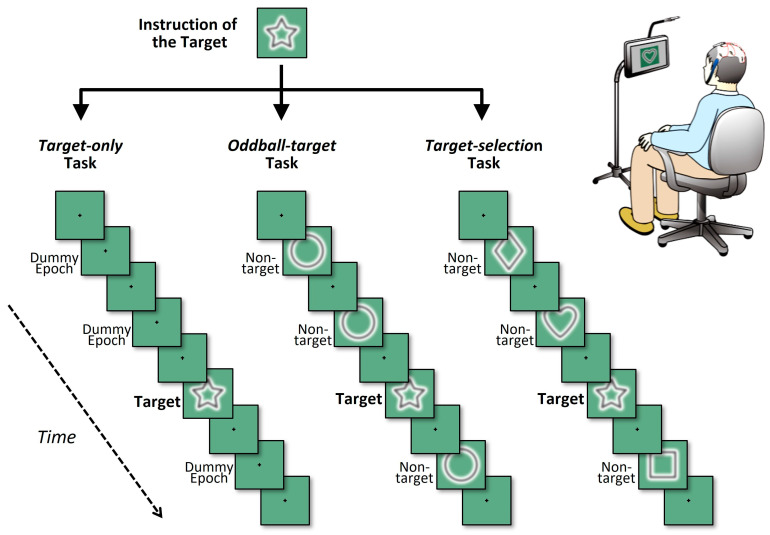
Schematic illustration of the three cognitive task conditions. Each panel depicts a partial sequence of one game, showing four visual stimuli (one target and three non-targets) interleaved at fixation-only intervals. In all tasks, each block consisted of eight stimulus intervals, within which one target stimulus and seven non-target or equivalent intervals were presented at fixed positions. Visual stimuli (shapes) appeared briefly between fixation spot-only intervals, which also defined inter-stimulus periods. The three tasks differed in terms of how the non-target intervals were handled. In the *target-only* task (**left**), only the target shape was presented, and all other intervals were replaced with a fixation spot (i.e., no shape was presented). In the *oddball-target* task (**middle**), the target appeared once among repeated presentations of an identical non-target shape. In the *target-selection* task (**right**), the target was presented among different non-target shapes, requiring shape discrimination in each trial.

The present study employed a Windows-based notebook PC equipped with an Intel^®^ Core™ i7-class CPU to control the presentation of the cognitive tasks. A 15-inch submonitor was positioned approximately 100 cm in front of the participant’s eyes, where the visual stimuli were displayed. Eight simple geometric shapes, including stars, hearts, diamonds, and circles, were used as visual stimuli, with different configurations depending on the task condition (Figure 1).

To systematically investigate neural responses under varying cognitive demands, three types of tasks were administered: *Target-only*, *Oddball-target*, and *Target-selection* [61]. In all tasks, eight types of simple geometric shapes (e.g., circle, square, triangle, star) were used as targets or non-targets. Across all tasks, a common temporal and structural format was used: each task consisted of eight “games,” and each game comprised six consecutive “blocks.” Within a game, one specific geometric shape was designated as the target, which remained constant throughout the six blocks. The participants were informed of the designated target shape prior to the start of each game. Each block included eight stimulus cycles (hereafter referred to as “trials”), in which a single target stimulus and seven non-target stimuli or equivalent dummy trials were presented in pseudorandom order. To ensure balanced cognitive processing, the temporal position of the target within each block was pseudorandomized with the constraint that it could not appear within the first two trials. For ERP analysis, target waveforms for each participant were obtained by averaging all target trials within each game condition. Given that each task consisted of eight games with six target trials per game, a total of 48 target trials per task (6 × 8) were integrated, providing a sufficient number of epochs to yield robust P300 signals.

In each trial, a shape was displayed for 375 ms, followed by a 250 ms interval containing only a small central fixation spot (a black crosshair), resulting in a stimulus cycle of 625 ms (1.6 Hz). The participants were instructed to maintain their gaze on the fixation spot throughout each game. A 10 s break was provided between the games. Thus, one game lasted approximately 30 s, and one task (eight games) required approximately 5 min and 10 s to complete. The total session duration, including two 5 min breaks between tasks, was approximately 25 min 30 s.

The specific characteristics of each task type are described below.

(1) Target-only task

This task involved repeated presentations of the target shape without any non-target stimuli, requiring participants to focus solely on mentally counting the number of target occurrences. Although no distractors were displayed, data segments corresponding to the timing of non-targets in other tasks were included as dummy epochs to preserve the same temporal structure. This task served as a cognitively simple control condition, as it did not require discrimination between targets and non-targets. This has been employed in previous studies as a baseline to isolate target-related neural responses without interference from distractors [22].

(2) Oddball-target task

This task involved infrequent presentation of the target shape within a stream of frequently presented identical non-target shapes, forming a classical oddball paradigm that is widely used in ERP research. In this paradigm, the target is a low-probability stimulus (12.5%) embedded among high-probability non-targets (87.5%), making it particularly effective in eliciting attention-related brain responses, such as the P300 component [62]. The oddball task has been extensively validated and remains one of the most frequently used methods in cognitive neuroscience, owing to its simplicity, high signal-to-noise ratio, and sensitivity to attentional and memory-related processes.

(3) Target-selection task

This task required participants to identify the target shape among heterogeneous distractors, making it the most cognitively demanding condition in this study. As the similarity between the target and non-targets increased, so did the task difficulty, necessitating sustained top-down attentional control. This paradigm aligns with tasks commonly referred to as sequential delayed matching-to-sample (DMTS) tasks, which are widely used in psychological research to investigate higher-order cognitive functions including working memory and selective attention [63]. However, such paradigms are less prevalent in ERP research because of the complexity and variability of the elicited neural responses.

Despite this, the *Target-selection* task is particularly relevant for brain–computer interface (BCI) applications that require users to make voluntary and flexible selections among multiple options based on internal intentions, such as choosing letters or emotional expressions. In this study, the *Target-selection* task was the most critical condition because it was used to evaluate the decoding accuracy of the EEG-based virtual switch. The average decoding performance and interindividual variability in this task served as key indicators of the system’s feasibility and potential for clinical application.

The *Target-selection* task was the most critical condition in this study because it was used to evaluate the decoding accuracy of the EEG-based virtual switch. The other two tasks, *Target-only* and *Oddball-target*, were positioned as control conditions to help interpret the effects of reduced cognitive function or attentional engagement observed during the *Target-selection* task.

These three tasks were intentionally designed to impose progressively increasing cognitive demands in the following order: *Target-only* < *Oddball-target* < *Target-selection*. Accordingly, it was hypothesized that decoding accuracy would inversely correlate with task difficulty and decrease as cognitive demand increases. This assumption reflects the core expectation of the study that increased attentional and discriminative requirements may impair the performance of ERP-based classification systems.

This relationship between task difficulty and attentional demands has been supported by previous findings on visual search and selective attention, which show that as the complexity of a visual search increases (e.g., more distractors or greater target-distractor similarity), the neural differentiation between target and non-target stimuli tends to diminish, making decoding more difficult [64,65].

### 2.4. EEG Recording

**Figure 2:**
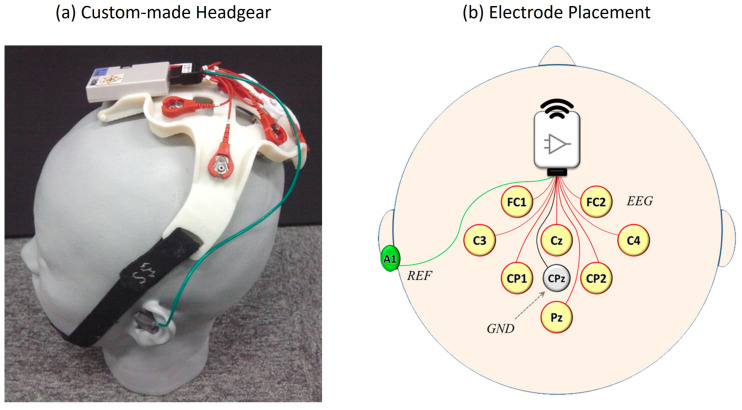
EEG recording system used in the experiment. (**a**) A custom-made 3D-printed headgear worn by a participant designed for a comfortable and secure fit using an adjustable chin strap. (**b**) Electrode placement diagram following the international 10–10 system, showing eight EEG channel (FC1, FC2, C3, Cz, C4, CP1, CP2, and Pz), reference (A1), and ground (CPz) electrode locations.

We utilized the EEG recording system from the Neurocommunicator to develop the Neurodetector and conducted experiments using these core technologies (Figure 2). The central component was a custom-made headgear manufactured using a 3D printer with resin material (Figure 2a). Three sizes (Small, Medium, and Large) were prepared to accommodate different head sizes and to ensure an optimal fit. Unlike conventional cloth EEG caps, this headgear was secured using a chin strap that was hooked under the tip of the chin, eliminating uncomfortable pressure on the scalp and avoiding any restriction on breathing.

EEG signals were recorded from eight scalp locations based on the international 10–10 system [66]: FC1, FC2, C3, Cz, C4, CP1, CP2, and Pz (Figure 2b). Stainless steel electrodes were used with gel-soaked cotton balls between the electrodes and scalp to ensure stable signal acquisition. These eight locations were labeled as Channels 1 through 8 for subsequent analysis. The electrode configuration was chosen to cover the central and parietal scalp regions, where ERPs, particularly the P300 component, are typically the most prominent [22,67].

The ground electrode was placed at the parietal site (CPz), and the reference electrode was attached to the left mastoid (A1) using a disposable solid gel electrode. Each participant completed one session per task for a total duration of approximately one hour. Throughout the sessions, no discomfort or complaints regarding the headgear were reported by the participants.

Raw EEG data were recorded at a sampling rate of 256 Hz and wirelessly transmitted to the same computer that controlled the task. The data were band-pass filtered from 0.2 to 30 Hz and down-sampled to 64 Hz. To extract a single-trial ERP waveform, the EEG was segmented into 1 s epochs, spanning from 200 ms before to 800 ms after stimulus onset, following standard ERP analysis procedures [20].

### 2.5. ERP Decoding

To evaluate the feasibility of the *EEG Switch*, we focused on identifying P300-like components elicited by target stimuli, a principle established in earlier P300-based communication systems [32]. Based on these observations, we investigated whether the subject’s intended target could be reliably decoded from EEG data during the target presentation period. In each game, the same object was presented six times as the target and interspersed with non-targets, allowing us to assess the classification accuracy under repeated exposure.

For decoding, we applied linear discriminant analysis (LDA) to transform the multidimensional ERP data into a single discriminant score. The input features for the LDA model consisted of ERP amplitudes sampled at 15 time points across eight EEG channels, resulting in 120 features. The LDA model was implemented using a standard formulation without explicit shrinkage regularization. Given the relatively low dimensionality (120 features) and sufficient number of training samples per subject, additional regularization was not deemed necessary. This high-dimensional approach avoids the arbitrary selection of time intervals or electrodes and is effective for single-trial EEG classification tasks [68].

The LDA model was trained using labeled data to separate the target and non-target trials. After training, the raw discriminant scores were linearly scaled such that the average score for the target trials was +1, and that for the non-target trials was −1. For each game, we calculated discriminant scores for the eight candidate options, with the option yielding the highest cumulative score identified as the predicted target. Classification accuracy was assessed using leave-one-out cross-validation, wherein the model was trained on all games except one, and tested on the held-out game. The final success rate for each participant was defined as the number of correctly predicted games divided by the total number of games (i.e., eight).

Through these processes, we evaluated whether each participant correctly selected the target using the *EEG Switch* in each game, that is, whether they were successful in that game. By aggregating these results across all the games in the task, we calculated the overall success rate for each participant. Since the visual stimuli consisted of easily identifiable geometric shapes and participants were assumed to mentally identify the correct target in each game, we referred to the proportion of correctly decoded targets, based on LDA discriminant scores, as the “decoding accuracy” of the target.

Quantitative data were analyzed using SPSS statistical software Version 17.0 (IBM Corp., Armonk, NY, USA). Two-way repeated-measures analysis of variance (ANOVA) was used to examine the effects of the task type and decoding method on decoding accuracy. Although some deviations from normality were observed, the sample size was sufficiently large (n = 40) to mitigate potential violations of the assumption of normality. Therefore, parametric analyses were deemed appropriate. Post hoc comparisons were performed using Bonferroni correction.

## 3. Results

### 3.1. An Example of Average ERP Analysis

**Figure 3:**
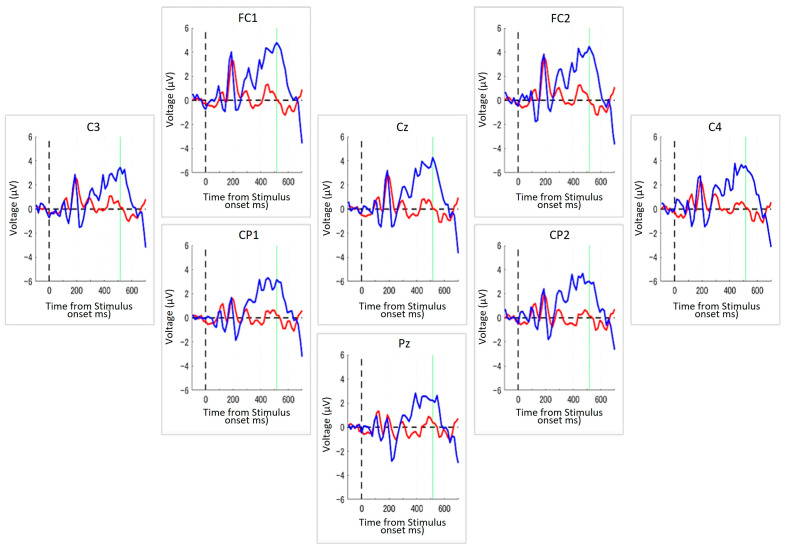
ERPs from a representative participant during the *Target-selection* Task. Each panel displays ERP waveforms recorded from one of the eight electrode positions arranged to reflect their approximate relative positions on the scalp, with top to bottom and left to right corresponding to the anterior–posterior and left-right orientations of the head, respectively. The blue line indicates the average ERP response to the target stimuli, and the red line represents the response to non-target stimuli. The dashed vertical line indicates the timing of stimulus onset, and the green vertical line denotes the peak latency identified from the grand average across all channels. The x-axis denotes the time (in milliseconds) from stimulus onset, and the y-axis represents the voltage (in μV). Across all electrode sites, a stronger positive deflection was observed in response to targets, typically peaking around 500 ms after the stimulus onset.

In this study, we evaluated the feasibility of using the *EEG switch* to perform cognitive tasks in 40 healthy adults. As an initial step, we analyzed individual differences in ERP responses to target and non-target stimuli during the task. Figure 3 illustrates a representative example of the averaged ERP waveforms from a single participant during the *Target-selection* task.

In this participant, a transient, non-selective response was observed around 150–200 ms post-stimulus onset across all electrodes, likely reflecting early perceptual processing. Following this initial component, a clear differentiation between target and non-target responses emerged. Specifically, the target stimuli elicited a pronounced positive deflection that peaked approximately 500 ms after stimulus onset. This positivity, characteristic of the P300 component, was broadly distributed across all the recording sites. By contrast, ERP responses to non-targets remained relatively flat following the early component, indicating a lack of strong cognitive engagement.

Although individual temporal dynamics varied among participants, this general pattern—an early non-selective response followed by a selective target-related positivity—was consistently observed, supporting the robustness of the *EEG Switch* in distinguishing cognitive responses. In addition, the success rates of the task completion were evaluated statistically. The mean success rate across all subjects was 76.2% (SD = 11.8%), which significantly exceeded chance-level performance by 50% (t(19) = 8.13, *p* < 0.0001).

### 3.2. Comparisons of Average ERPs Among the Three Tasks

**Figure 4:**
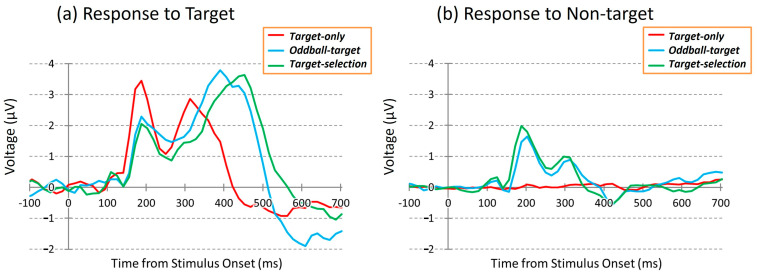
Grand-average ERP waveforms during the three tasks at the Cz electrode site. (**Left**): responses to target stimuli; (**Right**): responses to non-target stimuli. Red: *Target-only* task; Blue: *Oddball-target* task; Green: *Target-selection* task.

Figure 4 shows grand-average ERP waveforms elicited by target and non-target stimuli during the three cognitive tasks: *Target-only*, *Oddball-target*, and *Target-selection*. These waveforms represent the average across 40 participants and eight electrode sites (FC1, FC2, C3, Cz, C4, CP1, CP2, and Pz). As stated in *the Methods section*, the *Target-only* task required no discrimination, imposed minimal cognitive demand, and primarily engaged bottom-up attention. The *Oddball-target* task involved discrimination based on stimulus salience, requiring moderate cognitive effort with a continued reliance on bottom-up attention. In contrast, the *Target-selection* task required goal-directed discrimination and top-down attentional control, making it the most cognitively demanding task.

Biphasic ERP responses to the target stimuli were observed in all tasks. The first positive peak (~188 ms) was common across conditions but showed the largest amplitude in the *target-only* task. The second positive peak appeared at different times and with varying amplitudes depending on the task type, reflecting task-related modulation of neural activity. Despite similar spatial distributions of ERPs across tasks, waveform morphology differed in response to identical stimuli, suggesting that cognitive demands distinctly modulate the temporal dynamics of neural responses.

In contrast, ERP responses measured during dummy epochs of the *Target-only* task, corresponding to non-target trials, were minimal, as no visual stimuli were presented. In the *Oddball-target* and *Target-selection* tasks, however, non-targets elicited modest early positive deflections, particularly around the latency of the first peak. Interestingly, the responses to these non-targets also showed subtle differences between the two tasks, despite being evoked by the same set of visual stimuli, implying that the task context may modulate neural responses even to identical non-target events.

**Figure 5:**
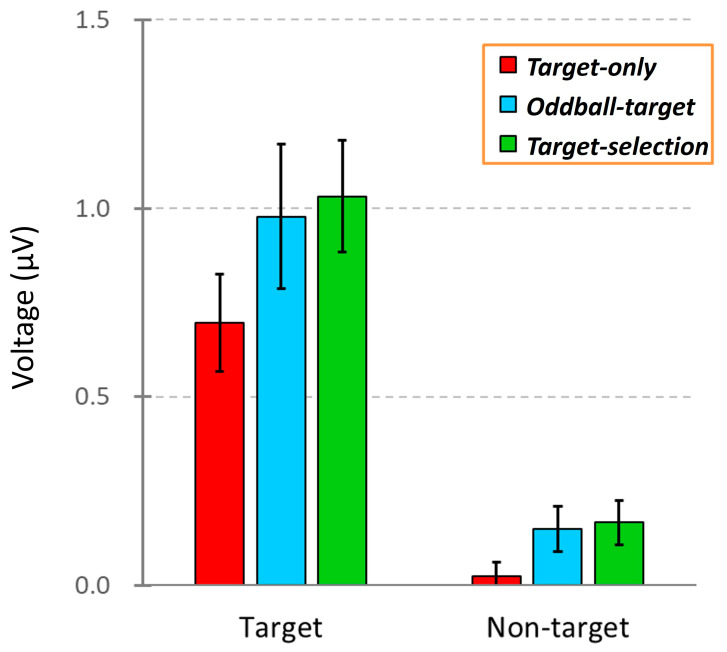
Comparison of ERP amplitudes across three task conditions: *Target-only*, *Oddball-target*, and *Target-selection*. Each bar represents the mean ERP voltage (±SEM) measured between 100 and 700 ms after stimulus onset in response to either the target or non-target stimuli. The data were averaged across the 40 participants.

To qualitatively compare how ERPs elicited by target and non-target stimuli (including dummy epochs in the *Target-only* task) differed across the three task conditions, we created Figure 5. As shown in Figure 4, the ERPs were averaged across all eight electrodes to highlight the overall response patterns while minimizing electrode-specific variability. In each task condition, the target stimuli evoked a clear positive deflection, whereas non-target stimuli elicited minimal responses. These patterns suggest systematic differences in neural processing between target and non-target stimuli depending on the task demands.

To test whether these differences were statistically significant, we conducted a two-way repeated-measures ANOVA with stimulus and task types as within-subject factors. ERP amplitudes were calculated as the mean voltage between 100 and 700 ms after stimulus onset based on data from 40 participants. The analysis revealed significant main effects of stimulus type (*F*(1, 39) = 30.11, *p* < 0.001) and task type (*F*(1, 39) = 12.22, *p* < 0.01) but no significant interaction between the two (*F*(1, 39) = 1.38, *p* > 0.05). Post hoc comparisons showed a significant difference between the *Target-only* and *Target-selection* tasks (*p* < 0.01). However, no significant differences were found between the *Oddball-target* task and the other two tasks, making it difficult to clearly establish an ordered gradient of task-related neural activity using conventional averaging methods.

While traditional ERP analyses provide qualitative insights into the relative differences across conditions, particularly over specific time windows where discriminative neural responses emerge, they do not quantify how accurately a given stimulus (e.g., target vs. non-target) can be decoded within each task condition. Moreover, the outcomes of such analyses can vary depending on the choice of time window, introducing the potential for interpretive bias.

In addition, given the substantial inter-individual variability in ERP waveforms, relying solely on comparisons to a grand-average waveform may obscure important individual characteristics. This may lead to under- or overestimation of the response strength and decoding performance, especially when an individual’s response pattern deviates from the group average.

Thus, when evaluating multivariate time-series data such as ERPs, conventional averaging methods have limited ability to assess the reliability of cognitive task performance. To address these limitations, we adopted a multivariate analytical approach, as described in the following section.

### 3.3. Example of Task Performance by EEG Switch

As stated earlier, it is not straightforward to estimate the degree of decision making involved in internal target selection merely by observing raw ERP waveforms. In the present study, we applied LDA to ERP patterns, as previously described, to quantify whether target-related neural activity during stimulus presentation could be reliably decoded.

**Figure 6:**
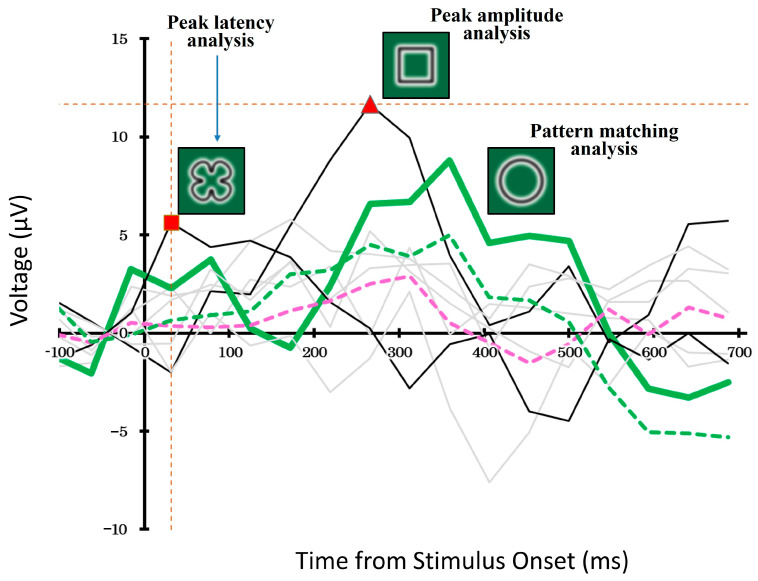
Example of stimulus decoding using ERP waveforms during the *Target-selection* task. The pattern-matching method (bold green line) selected the waveform most similar to a target-related ERP, whereas the peak amplitude and latency methods (black lines) chose stimuli based on amplitude or timing. Gray lines indicate unselected stimuli. Dashed lines represent average responses to targets (green) and non-targets (pink). The vertical orange dashed line and the red square indicate the latency peak detected by the latency analysis, while the horizontal orange dashed line and the red triangle indicate the amplitude peak detected by the amplitude analysis. These markers show the specific points at which the respective peak values were determined. This example highlights how decoding strategies can yield divergent results.

Figure 6 illustrates an example of stimulus decoding in a single trial that employs different decoding strategies. Specifically, we employed a pattern-matching method based on waveform similarity and compared it with two simpler methods, the peak amplitude and peak latency methods.

In the pattern-matching method, we compared the ERP waveforms evoked by each of the eight candidate stimuli and selected the waveform that best matched the typical target-related response pattern. The thick green line represents ERP response to the selected stimulus. In contrast, the black solid lines show the waveforms of the stimuli selected using the peak-amplitude and peak-latency methods. The remaining five non-selected stimuli are shown in gray.

The dashed green and pink lines represent the average ERP responses to targets and non-targets, respectively, calculated across the entire dataset using a cross-validation procedure. Although all three decoding methods operated independently, their outputs sometimes overlapped, and each method selected a different stimulus.

### 3.4. Progress of Decoding Accuracy Through Blocks by Pattern-Matching Method

To assess the performance of the pattern-matching method across different stages of the task, we analyzed the decoding accuracy block by block for all participants. Specifically, we computed the decoding accuracy not only for the final (sixth) block, but also for each of the preceding blocks (blocks 1 to 5). This allowed us to assess how quickly and reliably the system could distinguish the targets based on a limited number of repetitions.

**Figure 7:**
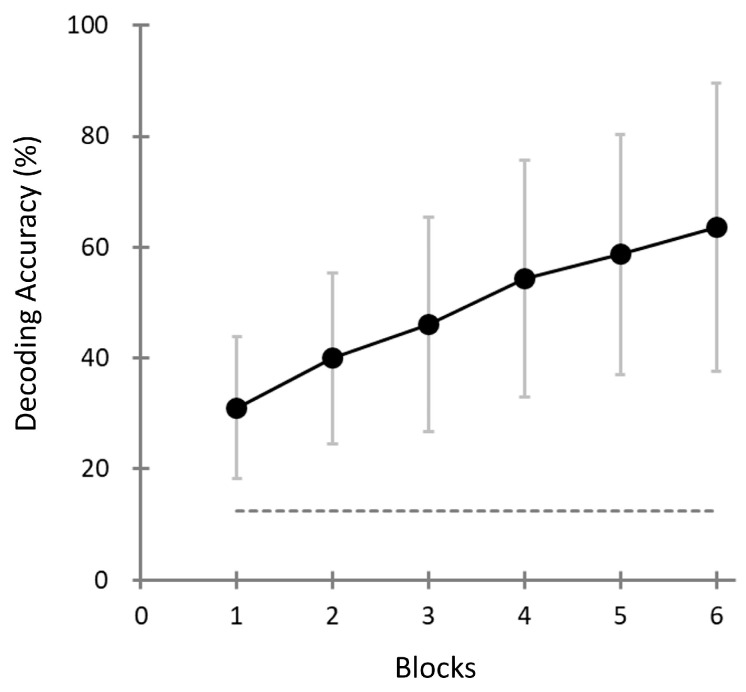
Decoding accuracy as a function of block number (i.e., number of cumulative stimulus presentation cycles) in the *Target-selection* task. Each data point represents the mean decoding accuracy across participants (N = 40) based on cumulative evidence up to that block (e.g., Block 3 reflects performance based on the first three stimulus trials). Error bars represent the standard deviation. The horizontal dashed line represents the theoretical chance level (12.5%) of the eight-choice task.

Figure 7 illustrates the progression of decoding accuracy across repeated stimulus blocks (1–6) in the *Target-selection* task. Each “block” corresponds to a cumulative evaluation based on an increasing number of stimulus presentations within a game (e.g., Block 3 reflects performance based on the first three trials). As shown, decoding accuracy steadily improved with increasing blocks, indicating the benefit of accumulating evidence over time. In the final block (Block 6), the mean decoding accuracy reached 63.6% (SD = 26%), which was significantly higher than the theoretical chance level of 12.5% for the eight-choice task (one-sample *t*-test, *t*(39) = 12.33, *p* < 0.001).

Although some participants achieved perfect decoding accuracy (i.e., 100%), the overall distribution of the decoding performance was statistically distinguishable from the ceiling effect. The Lilliefors test confirmed that the distribution did not deviate from normality (H = 0, *p* = 0.1769). Furthermore, a one-sample *t*-test against the theoretical ceiling value (100%) indicated that population-level performance was significantly lower (t(39) = −8.75, *p* < 0.001). These findings suggest that, while high individual performance was observed, the overall accuracy did not saturate, and performance improvements remain possible.

Notably, the decoding accuracy in the first block exceeded the chance level across participants, suggesting that sufficient discrimination may be possible with fewer repetitions. This finding indicates the potential of reducing task duration without compromising assessment reliability.

### 3.5. Comparison of Task Performance Across the Three Decoding Methods

**Figure 8:**
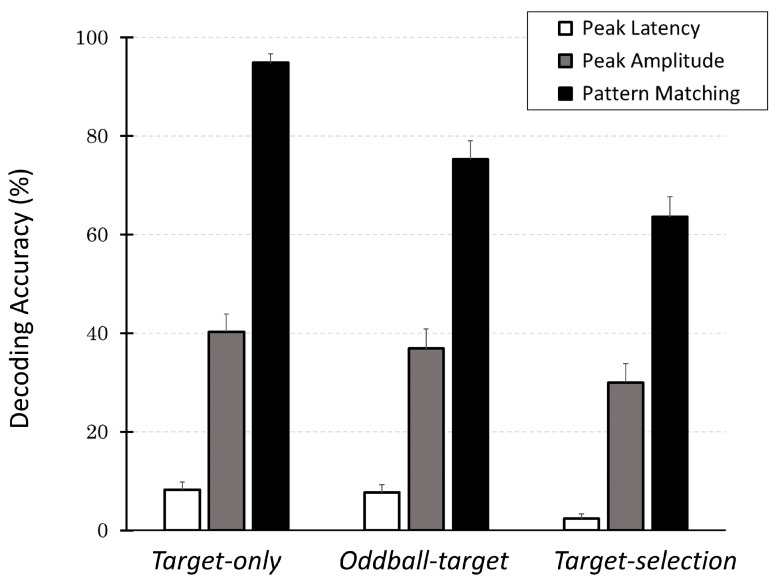
Decoding accuracy across three decoding methods (peak latency, peak amplitude, and pattern matching) across three cognitive tasks (*Target*-*only*, *Oddball*-*target*, and *Target*-*selection*). Each bar shows the mean percentage of correct identifications of the intended target (±SEM) for the 40 participants. The pattern-matching method consistently outperformed the other two methods for all the task types. In particular, its performance remained well above the chance level for 8-choice tasks (12.5%), even for the most demanding task. In contrast, peak-based methods fail to reliably decode the intended target. These results suggest that the pattern-matching method provides a robust and sensitive index for internal target selection, even with an increasing cognitive load.

To evaluate which decoding strategy most effectively identified internal target selections, we compared the decoding accuracies in the last block across the three methods and task conditions for all participants. Figure 8 provides a summary of the results.

Across all tasks, the decoding accuracy of the pattern-matching method was the highest, followed by the peak-amplitude and peak-latency methods. Among the decoding methods, decoding accuracy in the *Target-only* task was the highest, followed by the *Oddball-target* task, and the worst was the *Target-selection* task. These observations were supported by a two-way ANOVA, which revealed significant main effects of both task type (*F*(2, 78) = 28.08, *p* < 0.001) and decoding method (*F*(2, 78) = 318.20, *p* < 0.001), as well as a significant interaction between the two factors (*F*(4, 156) = 7.40, *p* < 0.001).

Post hoc multiple comparisons (with Bonferroni correction) further revealed that within the *Target-selection* task, decoding accuracy using the pattern-matching method was significantly higher than that using the peak-amplitude method (*p* < 0.001), which in turn was significantly higher than that using the peak-latency method (*p* < 0.001). Similarly, for the pattern-matching method, decoding accuracy was significantly (*p* < 0.001) higher in the *Target-only* task than in the *Oddball-target* task (*p* < 0.05), and both were significantly higher than in the *Target-selection* task (*p* < 0.05). These findings confirm that the overall trends remained consistent: pattern matching > peak amplitude > peak latency; *Target-only* > *Oddball-target* > *Target-selection*.

The results revealed that the pattern-matching method consistently outperformed the other two methods across all the task types. In contrast, the peak amplitude and peak latency methods showed much lower accuracies and failed to provide reliable decoding of the participants’ intended targets.

Importantly, the pattern-matching method demonstrated in this study provides a single quantitative index that reflects the clarity of target versus non-target discrimination based on multivariate EEG signals. These signals are otherwise difficult to interpret using conventional ERP features, such as peak amplitude or latency. This method captures subtle and distributed neural signatures of internal decision making by comparing the full spatiotemporal waveform to a learned template. Although a more detailed interpretation of these findings is presented in the following section, the current results underscore the utility of the EEG-based pattern-matching method as a robust and motor-independent neural marker for assessing top-down cognitive processes.

## 4. Discussion

### 4.1. Main Findings and Their Implications

This study investigated the feasibility of using an EEG-based virtual switch system for cognitive assessment by decoding ERPs during the *Target-selection* task. We emphasize again that the central goal of this study is not psychological theory testing but the development and baseline validation of the Neurodetector system as a technological solution for motor-independent cognitive evaluation. The central innovation of our approach is the application of a pattern-matching method to classify the multichannel ERP time-series signals. This approach was compared with conventional peak-based methods (i.e., peak amplitude and latency) for decoding attentional target selections. Across 40 healthy adult participants, we obtained three major findings.

(1) We demonstrated that the decoding accuracy in the *Target-selection* task was significantly above chance level (12.5%) in the final (6th) block and that the distribution of individual accuracy scores was broad enough to avoid a ceiling effect. This result confirms that, even after accumulating multiple stimulus presentations, performance remained unsaturated, thereby supporting the fundamental utility of the *EEG Switch* in detecting intentional target selection under realistic constraints.
(2) Our pattern-matching method, which is well suited for detecting subtle neural responses while accounting for individual variability, consistently outperformed both the peak amplitude and latency methods across all task types. This result demonstrates the robustness of the method in capturing the distributed neural signatures of attention-driven decision making.
(3) Decoding performance systematically reflected task difficulty: Accuracy was highest in the *Target-only* task, intermediate in the *Oddball-target* task, and lowest in the *Target-selection* task. This pattern corresponds to increasing demands on attentional processing from passive perception to bottom-up salience detection to top-down selection.

These findings have several important implications. First, the ability to achieve above-chance decoding accuracy using lightweight and easy-to-use EEG headgears supports the feasibility of practical deployment in clinical or home-based settings, even for individuals with impaired motor function. In this respect, the present results provide an important first step toward the development of motor-independent and scalable cognitive assessment tools. Future work will specifically target clinical populations, including MCI and dementia, to evaluate the diagnostic potential demonstrated here in healthy adults.

Second, the distinct performance profiles observed across the three tasks offer a valuable diagnostic utility. If decoding accuracy is impaired in the *Target-selection* task but preserved in the oddball task, the deficit may stem from a decline in top-down attentional control. If performance is also reduced in the oddball task, this may indicate a more generalized decline in attention, including in bottom-up processes. Furthermore, poor performance, even in the *Target-only* task, suggests an impairment in the reception or processing of visual information.

Taken together, these results suggest that this multi-task *EEG Switch* not only quantifies attentional capacity but may also help differentiate the underlying nature of cognitive decline by leveraging contrasts among attentional subsystems.

To validate this interpretation, we employed significance testing against theoretical chance levels, a standard and objective method widely used in multiclass BCI evaluations. Since the seminal P300 speller study by Farwell and Donchin (1988) [42], this approach has been consistently applied to assess whether classification performance reflects genuine brain-based signal processing or is merely due to random fluctuations [69,70,71,72,73]. For instance, Farwell and Donchin demonstrated classification accuracies up to 90% in a 36-choice task (chance level: 2.8%), while Acqualagna et al. (2016) reported 74% accuracy in a 9-choice task (chance: 11.1%, *p* < 0.01) [69]. One-sample *t*-tests provided a statistically grounded and interpretable benchmark for determining whether performance significantly exceeded chance. This is especially important in large-class BCI paradigms, where the relative gain over chance is a more meaningful indicator of efficacy than raw accuracy values.

While these studies primarily focused on communication-assistive applications aiming for maximal decoding accuracy, our study hypothesized that, at least in healthy individuals, decoding accuracy, particularly as measured in the final (6th) block of the *target-selection* task, would distribute moderately above chance without reaching ceiling levels. This balanced accuracy range would reflect meaningful variability in attentional capacity rather than perfect classification. Our experimental results support this hypothesis, confirming that the EEG Switch achieves sufficiently high but not saturated decoding performance, enabling the differentiation of attentional subsystems.

### 4.2. Significance of Cognitive Assessment by EEG Switch

Traditional cognitive assessment tools, such as the Mini-Mental State Examination (MMSE) [13] and Trail Making Test (TMT) [14], have been widely employed to detect cognitive decline, including early diagnosis of MCI and dementia [4,74]. The advantages of these tests lie in their simplicity, portability, and efficiency, as each typically requires less than 10 min to administer [75].

However, these tests have several limitations. MMSE outcomes can be influenced by non-cognitive factors, including language proficiency and literacy levels, thereby reducing cross-cultural reliability [74]. TMT is commonly employed to evaluate attention and executive function [75,76]; however, it requires hand-eye coordination and intact motor abilities, rendering it less suitable for patients with conditions such as stroke, brain injuries, neurodegenerative diseases, or orthopedic impairments [77].

Moreover, TMT completion times tend to increase with age, even among healthy individuals, owing to age-related decline in motor agility. Consequently, age-specific cutoff values have been established [75]. Nonetheless, completely disentangling cognitive and motor functions remains challenging because significant individual differences exist, even within the same age group [76].

In contrast, one of the primary advantages of the *EEG Switch* is its independence from motor responses, as it directly detects brain responses related to cognitive function. This makes it an attractive candidate for cognitive screening and monitoring in clinical populations where conventional assessments are infeasible. Furthermore, because it can be administered in a repeatable, objective manner, it holds promise for use in longitudinal tracking of cognitive status or intervention effects.

These viewpoints are primarily related to the performance of cognitive tasks that do not depend on motor functions, making up most of the functions of the BCI. In addition, this study has the advantage of focusing on ERPs as the signal sources. As mentioned above, ERPs are EEG components that reflect a momentary increase in attention, and it is thought that the response strength changes depending on how clearly the target and non-target are distinguished. Therefore, it is possible to objectively observe internal processes that cannot be judged only by whether a physical switch, such as a button, is operated, as a discrimination score based on the response strength of the ERP, and ultimately as decoding accuracy.

Of course, in the case of physical switches, differences in confidence in target selection are thought to be reflected in response latency; however, response latency is also strongly influenced by individual differences in movement agility and is subject to the constraints of brain/body disorders. Considering these factors, the characteristic expected from task performance using the *EEG Switch* is an index of cognitive function that combines the correct answer rate (after the motor element is removed) and the response latency.

This study obtained results that could be used as candidates for such an index. The average decoding accuracy of about 64% in six blocks per game may not be high enough for an eight-choice communication device, but considering the large variance (26%), it is important in that it allows us to determine where new test subjects are located relatively in this distribution.

However, what (other than motor function) causes this variance needs to be thoroughly examined in the future. First, the most “undesirable” factor is the error caused by electrical noise. It is almost impossible to eliminate such noise in the measurement of biosignals, not just EEG, but it is also possible to minimize its influence by collecting sufficient data in one test or repeating the test several times. However, a “desirable” factor is that variance is caused by individual differences in cognitive function.

To examine this possibility, in a previous study conducted on a different group of subjects, we found a significant negative correlation between performance on the *EEG Switch* task and the time required for the TMT task (especially the difficult TMT-B); that is, those with high *EEG Switch* performance were able to complete the TMT in a short time [78].

### 4.3. Advantage of EEG Switch by Pattern-Matching Method

This study employed a pattern-matching approach based on linear discriminant analysis (LDA) to decode ERP responses and implement virtual switching. LDA is a classical multivariate analysis technique that has been widely used to classify categorical outcomes in numerous scientific fields, contributing to the understanding of various natural and behavioral phenomena [79]. Despite its simplicity, LDA offers relatively high classification performance without the need for extensive hyperparameter tuning, making it particularly suitable for empirical research with limited data. Compared to more complex classifiers, such as neural networks or support vector machines, LDA is more transparent, interpretable, and less prone to overfitting when applied to small EEG datasets [80,81,82]. In BCI applications, LDA has been effectively applied to systems, such as the P300 speller [40], where the goal is to identify a target among multiple alternatives. However, because the fundamental classification is binary (target vs. non-target), these systems do not predict the target in a single analysis. Instead, a discriminant score was computed for each candidate and the candidate with the highest score was selected as the predicted target. This approach has been adopted in many BCI systems, including the Neurocommunicator [83].

In this study, LDA was used not as a dimensionality reduction method but as a supervised linear classifier to discriminate between target and non-target ERPs. Although LDA projects data onto a low-dimensional axis (typically one-dimensional in binary classification), this projection is optimized for class separation using label information. This is in contrast to methods such as principal component analysis (PCA), which reduces dimensionality without regard to class labels and may discard discriminative features essential to classification. Therefore, we did not apply PCA before LDA.

We also selected LDA over classifiers such as a Support Vector Machine (SVM) or Random Forest because of its interpretability, parameter-free implementation, and consistent outputs across subjects and tasks. These properties are critical in studies such as ours that emphasize comparisons across multiple cognitive conditions, where parameter tuning in models such as SVM could introduce analytical bias.

Our current study also followed this approach, but there are important differences in how LDA is used in communication and cognitive assessment systems. In communication-oriented BCIs (such as the P300 speller or the *Neurocommunicator*), a calibration or training session is first conducted to collect sufficient labeled data. A classification model is then built offline and applied in real time (in the test session) to predict the user’s intended target, with the results disclosed immediately to the user and potentially to the observers.

In contrast, cognitive assessment systems, such as the Neurodetector, do not reveal decoding outcomes during sessions. Instead, decoding is performed offline after the session, enabling an objective evaluation of how well the system can infer the target-related ERP responses. To ensure reliability, cross-validation methods, particularly leave-one-game-out cross-validation, were used to assess decoding performance. In this scheme, each task consisted of eight games; for each fold of cross-validation, data from seven games served as the training set (effectively acting as a “within-session calibration”), while the remaining one game served as the independent test set. This procedure was repeated for all eight possible hold-out games (7:1 split repeated eight times). Thus, unlike communication-oriented BCIs that require a dedicated pre-test calibration session, the Neurodetector embeds the calibration process into the same experimental run, avoiding additional session time while still preserving independent test data for unbiased performance estimation. This methodological difference ensures that the *EEG Switch* functions not as a feedback tool but as an evaluation instrument for the underlying cognitive processes.

ERPs are inherently small and noisy compared with ongoing EEG oscillations, muscle artifacts, and environmental noise. Therefore, conventional methods based on the peak amplitude or latency often fail to reliably extract target-related signals. In contrast, the pattern-matching method compares the full spatiotemporal ERP waveform with a template, enabling the detection of subtle, distributed differences between targets and non-targets. Rather than fitting a model to the entire dataset—which could artificially inflate performance estimates—we used cross-validation to partition each participant’s data into separate training and test sets. This approach not only prevents overfitting, but also mimics the constraints of real-time EEG Switch operation, where decoding must be based only on data observed up to that point. Thus, the analysis framework provides performance estimates that are both statistically robust and directly interpretable as indicators of cognitive task execution under realistic BCI conditions.

Although the accuracy of the *EEG Switch* decoding may not exceed that of physical switches, this property—moderate success rates with high variance—makes it suitable for assessing individual differences. In physical switch tasks, the success rate of the *Target-selection* task was nearly 100% [76], and intra-individual reaction time was the key variable. However, in individuals with motor impairments, reaction time may reflect physical constraints more than cognitive function. Therefore, *EEG Switch* offers a more equitable measure of attentional capacity across individuals with different physical abilities.

Importantly, system variability can be leveraged to quantify the effects of training or intervention, either within individuals or across populations.

### 4.4. Significance of Target Selection Task

In this study, the *Target-selection* task was introduced as the most cognitively demanding of the three cognitive tasks. Unlike the *Target-only* and *oddball-target* tasks, which rely primarily on perceptual recognition and bottom-up attentional capture, respectively, the *Target-selection* task requires the participant to actively direct top-down attention based on internalized instructions and selectively identify a target shape among heterogeneous distractors. This form of selective attention plays a fundamental role in everyday life, as it underpins goal-directed behavior, such as choosing objects, filtering distractions, and interpreting complex visual scenes. A decline in top-down attentional control is closely associated with a reduced quality of life, especially in older adults and individuals with cognitive impairment.

To ensure that the participants, particularly those with motor or cognitive limitations, could reliably perceive and process the presented stimuli, we adopted a temporally guided card-flip-like stimulus presentation format. Each stimulus appeared individually in succession, similar to a flashcard, minimizing visual overload while enabling precise ERP time-locking. Although this method increases the testing duration, it improves stimulus clarity and attentional allocation control. Importantly, such stimulus presentation paradigms have long been utilized in cognitive psychology as versatile tools for investigating various cognitive domains, including attention, working memory, and decision making. Classic reviews and empirical studies support their effectiveness as general-purpose behavioral paradigms for cognitive task design [84,85,86].

From a neurophysiological perspective, the temporally discrete nature of this paradigm is well suited for ERP measurements. While most ERP studies in clinical and aging populations rely on the oddball paradigm to elicit robust P300 components [22,23], some studies have demonstrated the feasibility of ERP measurement during the *Target-selection* task [87,88], offering insight into top-down control and visual attention mechanisms. Nevertheless, the use of target selection paradigms in ERP-based cognitive assessments, especially for early detection of cognitive decline in older adults, remains surprisingly limited. This reflects a bias toward the oddball task in ERP-based screening tools, leaving a gap in differentiating specific subcomponents of attention.

By incorporating the *Target-selection* task into the Neurodetector, the present study demonstrated its potential to complement rather than replace the oddball paradigm. While the oddball task predominantly captures automatic, bottom-up attention, the target selection task selectively engages in intentional top-down processes. Their combination allows for a more nuanced assessment of attentional mechanisms, and can help identify the source of cognitive difficulty in individuals with impaired performance.

Furthermore, by including the *Target-only* task with minimal attentional demand, the system offers a structured framework for inferring the level and nature of the impairment.

(1) Impaired performance only in the Target-selection task may indicate top-down attentional deficits.
(2) Poor results in both the *Target-selection* and *Oddball-target* tasks may suggest broader attentional dysfunction, including deficits in bottom-up attention.
(3) Impairments across all three tasks may reflect fundamental perceptual or vigilance deficits.

Our test battery (*Target-only*, *Oddball-target*, and *Target-selection* tasks) not only enables multilevel cognitive profiling, but also provides a foundation for constructing a test battery for cognitive function assessment based on BCI. The test design and structure of the Neurodetector have been recognized as novel and practically significant, as evidenced by the acquisition of patents in both Japan [89] and the United States [90].

### 4.5. Limitations

While the results of this study highlight the feasibility and potential of the *EEG Switch*, several limitations should be acknowledged to contextualize these findings. First, it included only healthy adult participants, most of whom were under 65 years of age. Consequently, the generalizability of these results to elderly individuals or those with cognitive impairment remains limited. Future research should include clinical populations, such as older adults with MCI or dementia, to evaluate the true diagnostic sensitivity and specificity of the *EEG Switch* in at-risk groups [17,18,19].

Second, although the pattern-matching method yielded a higher decoding accuracy than conventional peak-based approaches, the system still required the accumulation of six blocks to ensure robust performance. However, the total time to complete the current protocol, which includes eight games per task and six blocks per game, is approximately 25 min and 30 s, respectively, including breaks. The fatigue ratings collected after each task indicated no major complaints among the healthy adults. Nevertheless, for populations with cognitive or physical limitations, such as older adults or individuals with disabilities, it may be beneficial to reduce each session to three blocks per game and to split each task into two separate sessions with extended breaks. Importantly, as neurocognitive assessment does not necessarily require perfect accuracy akin to communication systems, reducing the block count per game while maintaining a sufficient number of games (e.g., 16 games with three blocks each) could maintain the diagnostic validity of the system while reducing the burden. Promising efforts are being made to improve single-trial decoding using machine learning and deep learning algorithms, which may ultimately enhance the practicality and responsiveness of ERP-based BCIs in real-world applications [91,92].

Third, the *EEG Switch* uses a compact and user-friendly headgear; it requires basic operator training and environmental control (e.g., impedance checking, artifact minimization, and signal quality assurance). These requirements may hinder deployment in resource-limited settings such as home visits. However, recent advancements in automatic EEG setup systems and dry electrode technologies have shown promise in overcoming these barriers [93].

Finally, although the interpretation of decoding accuracy based on attentional subsystems (e.g., top-down vs. bottom-up control) is theoretically motivated, the neurophysiological specificity of these distinctions remains to be empirically validated. Complementary methods, such as fMRI, pupillometry, and simultaneous eye tracking, can provide converging evidence to verify whether differences in EEG decoding performance truly reflect distinct cognitive mechanisms [94].

These limitations do not undermine the core findings of this study but rather identify future avenues for enhancing the system’s clinical utility, scalability, and mechanistic interpretability.

### 4.6. Future Directions

Building on these limitations, several avenues for future research and development have been proposed to enhance the applicability and functionality of the *EEG Switch*.

#### 4.6.1. Optimizing Block and Game Configuration for Efficient Assessment

The *EEG Switch* currently relies on six-block accumulation to ensure robust decoding of target-related ERP signals. Reducing the number of blocks per game, for example, from six to three, may offer a practical trade-off between accuracy and assessment time, particularly for screening scenarios where near-perfect accuracy is not required. Furthermore, if robust decoding can be achieved using fewer blocks, ideally, a single block, this would open new possibilities not only for streamlining cognitive assessment protocols but also for developing interfaces that are fully interchangeable with conventional physical switches. Such advancements would allow deployment of a broader range of cognitive tasks, including more complex executive function tests, using EEG-based BCI systems. Furthermore, improvements in single-trial decoding performance could enhance the utility of this system not only for one-time assessment, but also for repeated use in communication support systems, where multiple iterations are typically required.

Another potential direction is to increase the number of games per task (e.g., from 8 to 16) while reducing the number of blocks per game (e.g., from six to three). This configuration may offer a fine-grained assessment of performance without increasing the overall session time, and may better reflect continuous cognitive states while maintaining participant comfort and focus.

#### 4.6.2. Incorporating Few-Shot Learning Techniques

Recent advances in Few-Shot Learning (FSL) suggest the possibility of training robust classification models with limited data samples [95,96,97]. Although our current implementation uses LDA, which is computationally efficient and completes decoding in less than a minute, FSL techniques can further reduce the need for lengthy calibration procedures or large training datasets. These approaches are particularly advantageous when working with populations for which long EEG calibration sessions are burdensome, such as individuals with cognitive decline or reduced attention spans.

Incorporating meta-learning or prototype-based classification frameworks may enable generalization from a small number of labeled EEG trials, thereby enhancing their usability in clinical settings.

#### 4.6.3. Integration of Compressed Sensing

Compressed sensing is another promising framework that can be integrated into future iterations of the *EEG Switch*. Compressed sensing techniques, such as Adaptive Step size Forward–Backward Pursuit (AS-FBP) [98], and their application in noisy signal domains [99] offer additional possibilities for reducing training time and enhancing signal recovery. Although not employed in the current implementation, such approaches may be incorporated into the *EEG Switch* in the future to improve computational efficiency and decoding performance, particularly for real-time use.

#### 4.6.4. Clinical Validation in Older Adults with Cognitive Decline

To validate the diagnostic potential of the *EEG Switch*, future research should examine its performance in older adults with varying levels of cognitive function, including those diagnosed with MCI or dementia, compared with healthy age-matched controls. If a systematic decline in task performance is observed in the order of healthy > MCI > dementia, the *EEG Switch* may be established as a neurophysiological biomarker independent of motor output. Correlation with standard neuropsychological assessments is essential to support its clinical relevance. Large-scale data collection is the key goal.

Our system’s short setup time (less than one hour), user-friendly headgear, and intuitive graphical interface make it feasible for non-expert operators to acquire high-quality data, facilitating widespread deployment. Although the current implementation used eight electrodes, preliminary analysis suggested that the decoding performance remained at a promising level when only five central electrodes (FC1, FC2, Cz, CP1, and CP2) were used. While optimizing the electrode configuration was not the main focus of this study, these findings support the feasibility of simplifying the system for broader deployment and will help us build a normative database for elderly individuals and track longitudinal changes that may predict cognitive decline or response to treatment.

#### 4.6.5. ERP-Based Neurofeedback with Neurotrainer

Parallel to the assessment-focused *EEG Switch*, we are also developing a gamified cognitive training system called *Neurotrainer* [100], which uses BCI-based real-time feedback to promote engagement and cognitive enhancement. Unlike the Neurodetector, which emphasizes passive evaluation, the Neurotrainer allows participants to actively control game play using their brain signals.

Whereas the *EEG Switch* currently relies on six-trial accumulation, the Neurotrainer aims to achieve single-trial decoding, thereby enabling adaptive training protocols tailored to real-time cognitive states. Traditional neurofeedback systems have primarily focused on oscillatory EEG (e.g., alpha and beta), especially through interventions targeting alpha band activity for cognitive and behavioral modulation [101]. In contrast, ERP-based neurofeedback remains underexplored, partly because of technical limitations that hinder reliable single-trial decoding [102]. Our ongoing research will examine whether the Neurotrainer can prevent or delay cognitive decline when used regularly, and whether the Neurodetector can reliably track ERP-based biomarkers throughout training.

#### 4.6.6. Expanding the Target Population and Social Impact

Beyond aging and dementia, the *EEG Switch* may be applied to other populations, including individuals with acquired brain injuries, developmental disorders, or severe motor disabilities. This could also support the prevention of the disuse syndrome in bedridden patients. This broad applicability highlights the versatility and social importance of EEG-based cognitive technologies.

## 5. Conclusions

This study demonstrated the feasibility of using a virtual *EEG Switch* based on the pattern-matching method of ERP signals as a motor-independent tool for cognitive assessment. Among 40 healthy adults, we showed that decoding performance significantly exceeded chance levels and reflected both individual differences and task difficulty. The pattern-matching approach consistently outperformed conventional peak-based methods and enabled the meaningful evaluation of attentional processes across tasks with varying cognitive demands. For example, in the *Target-selection* task, the decoding accuracy averaged 63.6% (SD = 26%), which was far above the chance level of 12.5% (*p* < 0.001), demonstrating robust task differentiation. By integrating three tasks, the *Target-only*, *Oddball-target*, and *Target-selection*, we created a framework that allows not only the quantification of cognitive performance, but also the potential differentiation of specific cognitive deficits, such as impairments in top-down versus bottom-up attention. The use of a lightweight EEG headgear and short task duration further support the potential of the system for practical deployment in clinical and home-based environments. In conjunction with our ongoing development of the Neurotrainer, the present study lays the foundation for a unified system that assesses and enhances cognitive function through EEG-based interfaces. These findings mark an important step toward the development of accessible, objective, and scalable neurocognitive technologies for aging societies and individuals with motor or cognitive impairments.

## Data Availability

Data supporting the findings of this study are not publicly available because of their involvement in ongoing research projects and planned future publications.
